# A Novel Human Tectonin Protein with Multivalent β-Propeller Folds Interacts with Ficolin and Binds Bacterial LPS

**DOI:** 10.1371/journal.pone.0006260

**Published:** 2009-07-16

**Authors:** Diana Hooi Ping Low, Zhiwei Ang, Quan Yuan, Vladimir Frecer, Bow Ho, Jianzhu Chen, Jeak Ling Ding

**Affiliations:** 1 Computational and Systems Biology, Singapore-MIT Alliance, Singapore, Singapore; 2 Department of Biological Sciences, National University of Singapore, Singapore, Singapore; 3 Laboratory of Molecular Biostructural and Nanomaterial Modeling, AREA Science Park, Trieste, Italy; 4 Cancer Research Institute, Slovak Academy of Sciences, Bratislava, Slovakia; 5 Department of Microbiology, National University of Singapore, Singapore, Singapore; 6 Koch Institute for Integrative Cancer Research and Department of Biology, Massachusetts Institute of Technology, Cambridge, Massachusetts, United States of America; Charité-Universitätsmedizin Berlin, Germany

## Abstract

**Background:**

Although the human genome database has been completed a decade ago, ∼50% of the proteome remains hypothetical as their functions are unknown. The elucidation of the functions of these hypothetical proteins can lead to additional protein pathways and revelation of new cascades. However, many of these inferences are limited to proteins with substantial sequence similarity. Of particular interest here is the Tectonin domain-containing family of proteins.

**Methodology/Principal Findings:**

We have identified hTectonin, a hypothetical protein in the human genome database, as a distant ortholog of the limulus galactose binding protein (GBP). Phylogenetic analysis revealed strong evolutionary conservation of hTectonin homologues from parasite to human. By computational analysis, we showed that both the hTectonin and GBP form β-propeller structures with multiple Tectonin domains, each containing β-sheets of 4 strands per β-sheet. hTectonin is present in the human leukocyte cDNA library and immune-related cell lines. It interacts with M-ficolin, a known human complement protein whose ancient homolog, carcinolectin (CL5), is the functional protein partner of GBP during infection. Yeast 2-hybrid assay showed that only the Tectonin domains of hTectonin recognize the fibrinogen-like domain of the M-ficolin. Surface plasmon resonance analysis showed real-time interaction between the Tectonin domains 6 & 11 and bacterial LPS, indicating that despite forming 2 β-propellers with its different Tectonin domains, the hTectonin molecule could precisely employ domains 6 & 11 to recognise bacteria.

**Conclusions/Significance:**

By virtue of a recent finding of another Tectonin protein, leukolectin, in the human leukocyte, and our structure-function analysis of the hypothetical hTectonin, we propose that Tectonin domains of proteins could play a vital role in innate immune defense, and that this function has been conserved over several hundred million years, from invertebrates to vertebrates. Furthermore, the approach we have used could be employed in unraveling the characteristics and functions of other hypothetical proteins in the human proteome.

## Introduction

Advances in sequence genomics have resulted in an accumulation of a large number of protein sequences derived from genome sequences. Although the human genome database has been completed a decade ago, about 50% of the human proteome still remains hypothetical as their functions are unknown [1]. The elucidation of the functions of these hypothetical proteins can lead to additional protein pathways and revelation of new cascades, thus completing our fragmentary knowledge on the proteome complex. Furthermore, information on the network of protein–protein interactions will increase logarithmically. New hypothetical proteins may serve as disease markers and pharmacological targets.

The prime targets for the discovery of functional proteins are those which show homology to counterparts in lower species by way of sequence similarities and domain conservation. An alternate approach is to examine the proteins of invertebrates that do not have homologs in the vertebrate system. One example of such a group of proteins is the Tectonin domain-containing proteins in humans. Tectonin domain containing proteins, which belong to a subclass of proteins of the larger β-propeller family, have thus far only been studied in the fish, horseshoe crab, slime mold and sponge [2]–[5]. Tectonin domains were first reported in the Tectonins I and II proteins of the slime mold, *Physarum polycephalum*. The Tectonins I and II were characterized to have repeats of Tectonin domains [2]. Because the proteins are located at the surface of this organism, where they were postulated to scavenge food including bacteria, the Tectonin proteins have been speculated to function as bacterial sensors. Tachylectin-1 in the horseshoe crab, *Tachypleus tridentatus*, also has Tectonin domain classification [4], [6], and was shown to be able to bind bacterial lipopolysaccharide (LPS). Study on the Tectonin protein, LEC_SUBDO, of the sponge, *Suberites domuncula*, also revealed a possible LPS-binding function [3]. A recent investigation on the galactose-binding protein (GBP) in the horsehoe crab (*Carcinoscorpius rotundicauda*), a protein consisting of only 6 Tectonin domains revealed that the Tectonin domains function to differentiate host from pathogen and simultaneously bridge a host-pathogen interactome (Low et al., unpublished).

An exhaustive search in the databases for vertebrate proteins failed to reveal any potential homologs with significant sequence similarity, indicating that perhaps these Tectonin domain-containing proteins (henceforth referred to as Tectonin proteins) have evolved through the species, although more recently, other proteins with Tectonin domains are being uncovered, for example, the human leukolectin (GenBank Accession No. ACM77812). There are many examples of other families of meiosis-related proteins, kinetochores, cell gap contacts and nuclear pore complexes which show no homology at the primary amino-acid sequence level. However, they hint at the conservation of their domain architecture organization. Furthermore, the three-dimensional structure of functionally important domains in proteins in the budding yeast, nematode, Drosophila, Arabidopsis, and human have been conserved [7]–[11]. Here, we have used several databases like SCOP, CATH, SMART, which also employ domain and secondary structure classification for structure sorting and function prediction, to search for β-propeller structures and possibly distance relationships by domain conservation. This is especially useful when searching for related proteins with low sequence homology or when sequences have diversified through evolution from the invertebrates to the mammals. We thus seek to identify Tectonins in the vertebrates, and compare their domain architecture and function with ancient homologs from the invertebrates in order to gain insights into their functional conservation in the vertebrates, particularly in view of host-pathogen interactions.

By using known invertebrate Tectonin proteins, we performed domain- and conserved position-specific iterated sequence searches, and identified a potential human homolog, which we dubbed the hTectonin. We also discovered that the domain architecture of hTectonin is well conserved throughout the different species, suggesting that it is an important functional protein. Sequence motif analysis, and prediction of the secondary and tertiary structures suggests that hTectonin is a β-propeller protein, in accordance to the definition of the Tectonin domain. Specifically, only the Tectonin domains of hTectonin were found to interact with the fibrinogen-like domain of M-ficolin, an important complement initiator [12]. In addition, the hTectonin domains 6 and 11 also exhibited LPS-binding properties. The specificity of recognition of LPS by certain Tectonin domains is consistent with the invertebrate Tectonins such as the limulus GBP. We suggest that hTectonin forms a β-propeller structure involved in protein-protein interaction amongst host proteins and also in pathogen-detection, thus playing a vital role in bridging the host immune defense proteins to the invading pathogen, and this phenomenon is probably conserved over a vast number of organisms.

## Results

### hTectonin identified from the human genome database – *a hypothetical protein*?

**Figure 1:**
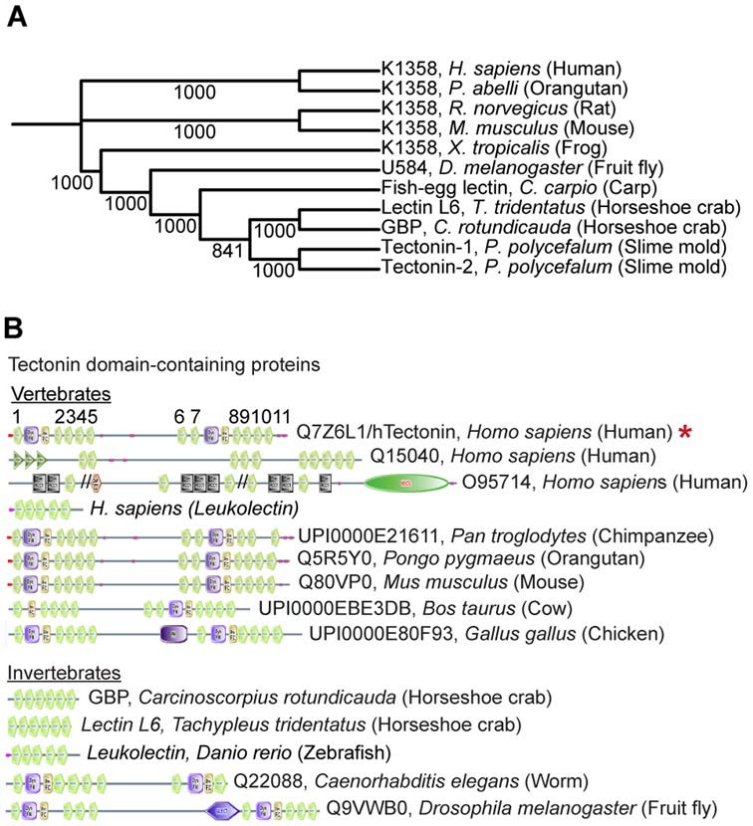
hTectonin is distantly related to the invertebrate Tectonins. (A) The phylogenetic tree constructed after a PSI-Search query using the invertebrate Tectonins revealed K1358 family of proteins as closely related Tectonin domain containing proteins in the mammals and also in lower species like the frog. The numbers at the nodes are an indication of the level of confidence for the branches as determined by bootstrap analysis (1000 bootstrap replicates). (B) Bioinformatics domain analysis utilizing SMART [22], [23] shows existence of Tectonin domain-containing proteins both in invertebrates and vertebrates from the horseshoe crab lectins, worm, up to humans. Of interest in this study is the protein hTectonin (red asterisk) which appear to have homologues in other species as well, for example in *P. troglodytes* (chimpanzee), *P. pygmaeus* (orangutan), *M. musculus* (mouse), *G. gallus* (chicken), *C. elegans* (worm) and *D. melanogaster* (fruitfly).

**Figure 2:**
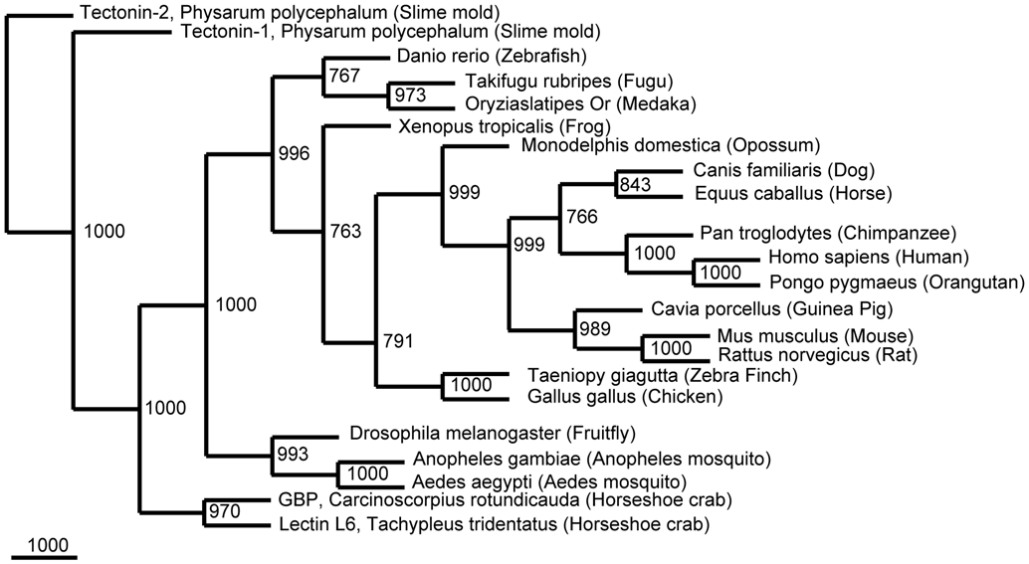
hTectonin gene is widespread across many species. The phylogenetic tree of hTectonin homologues constructed by primary sequence similarity shows its prevalence and conservation among a vast number of different species, right down to the worm, *C. elegans*. The human hTectonin protein was used as a query sequence in BLAST. Top hits were then compiled and multiple sequence alignment based on a guide tree was done using CLUSTALW [32] and the alignment was edited with Jalview [33]. The tree was constructed using the neighbour joining algorithm of the PHYLIP package. The numbers at the nodes are an indication of the level of confidence for the branches as determined by bootstrap analysis (1000 bootstrap replicates).

In mammals, the identity and role of proteins with Tectonin domains are unknown. Those identified or studied in the invertebrates [2]–[4], [6], [13]–[21] as well as the first vertebrate, fish, exhibit immune defense properties. Here, we sought to examine whether the Tectonin domains are structurally and functionally conserved in the mammals. A position-iterated search using known Tectonin domain-containing proteins in the invertebrates revealed a family of vertebrate Tectonin proteins to be distantly related (Figure 1A). This includes the human protein, Q7Z6L1, which is one of 3 human hypothetical proteins (GenBank Accession No.s: Q7Z6L1, Q15040 and O95714) that contain the Tectonin domain architecture [22], [23], when a domain architectural search was done on Tectonin domain-containing proteins. Q7Z6L1 codes for a predominantly Tectonin domain-containing protein (Figure 1B), suggesting that the domains probably form an essential part of the molecular structure and play a vital role. Furthermore, the high architectural homology of Q7Z6L1, from the slime mold to the human, suggests its evolutionary conservation and functional significance (Figure 2). We thus selected Q7Z6L1 which codes for ‘hTectonin’ for molecular expression, and further structural and functional analyses.

### hTectonin consists of β-propeller secondary structure

**Figure 3:**
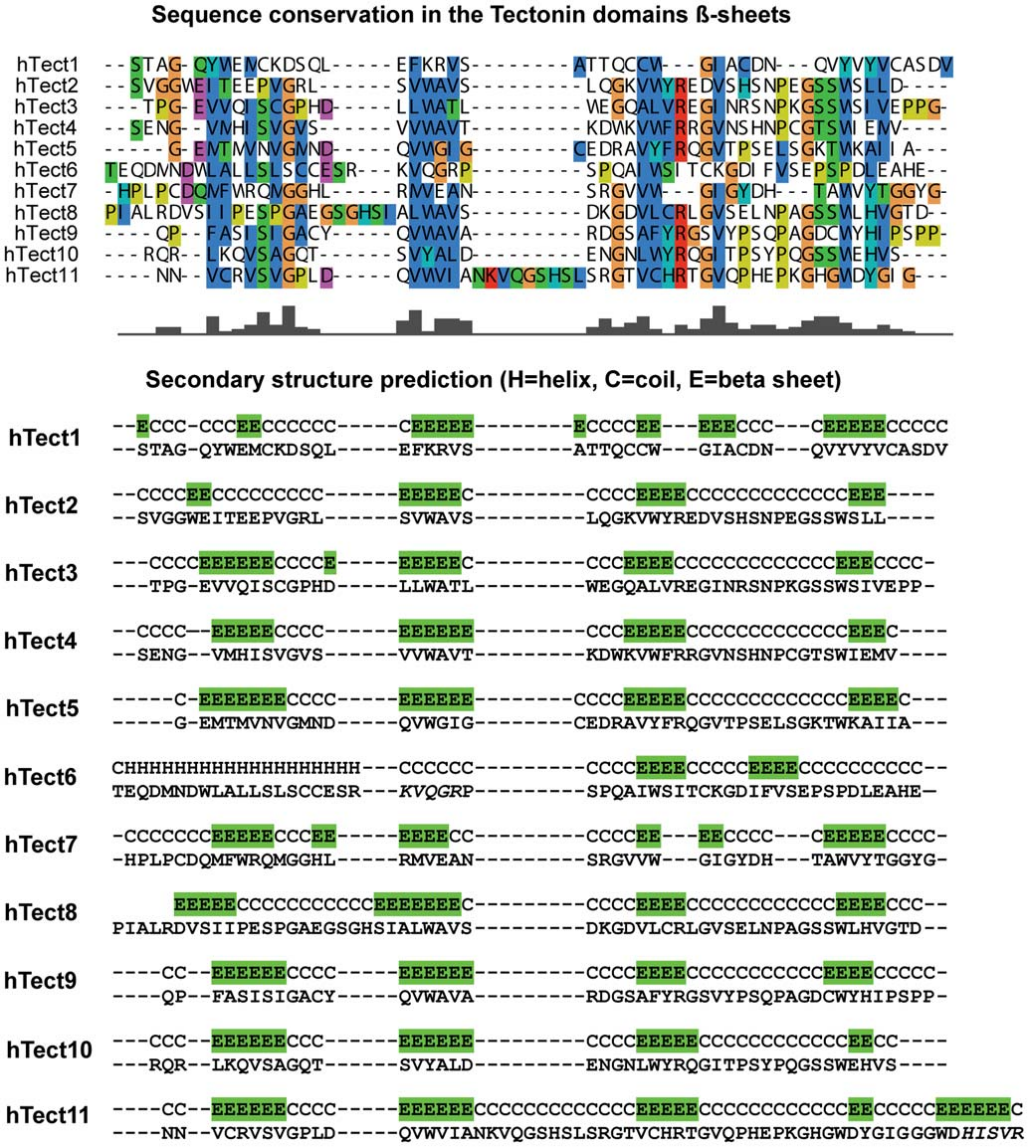
hTectonin forms β-sheets in its Tectonin repeats. CLUSTALW alignment of the individual Tectonin domains and PSIPRED secondary structure prediction indicates that the 11 Tectonin domains of hTectonin contain 4 highly conserved repeats that form β-strands (highlighted in green), a motif that is characteristic of the β-propeller fold. The LPS-binding motifs are in red font. E, β-sheet; C, Coil; H, Helix.

From the multiple sequence alignment (MSA) of the Tectonin domains, we confirmed a pattern of sequence repeats of 40 to 50 residues in length, which is a unique characteristic of β-propellers [9], [10]. In addition, secondary structure prediction of hTectonin by PSIPRED [24], [25] predicted these conserved repeats to form the β-strands of a β-sheet topology, consistent with β-propeller architecture (Figure 3).

### hTectonin interacts with ficolin through its Tectonin domains

**Figure 4:**
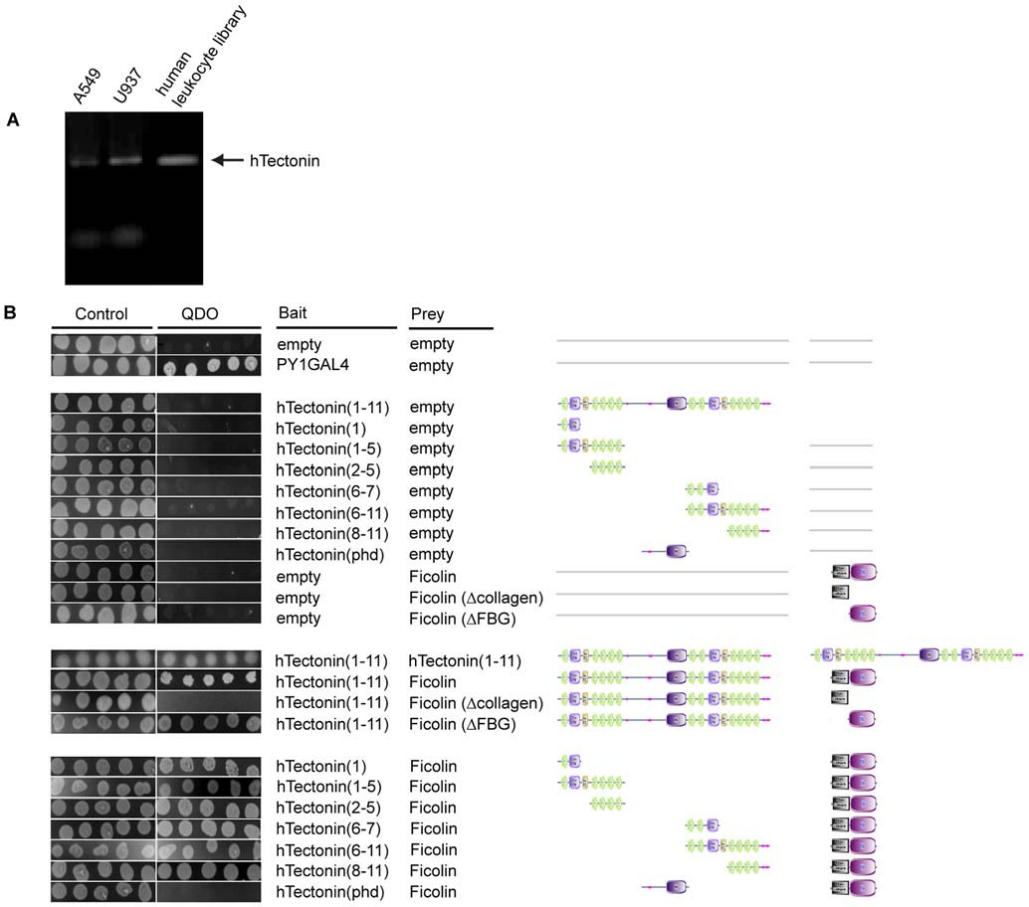
hTectonin exists and interacts with immune-related genes. (A) hTectonin cDNA is found in the human T cells (A549), monocytes (U937) and leukocytes. (B) hTectonin interacts with ficolin. Yeast 2-hybrid shows that hTectonin interacts (i) with itself, suggesting the possibility of oligomerization, as observed in other beta-propeller proteins; and (ii) with ficolin, a human complement protein. Furthermore, interaction with ficolin specifically occurs through the Tectonin domains of the hTectonins. This demonstrates a possible functional conservation of Tectonin domains since the Tectonin domains of GBP (horseshoe crab Tectonin lectin) was shown to interact with carcinolectin-5, a homologue of ficolin [26].

Based on our observations that a Tectonin protein, GBP (GenBank Accession No. AAV65031.1), interacts with two complement proteins, C-reactive protein (CRP) and carcinolectin (CL5), and is therefore immune-related, we reasoned that the hTectonin might play a similar role in immune defense. We tested and showed that the hTectonin gene is expressed in the human T cell line (A549), monocytes (U937) and the human leukocytes (Figure 4A), corroborating its immune relevance. Based on the rationale that (i) hTectonin is an architectural homolog of GBP and (ii) as a pathogen pattern-recognition receptor, GBP interacts with CL5 [26], which is a homolog of the human ficolin, we performed yeast 2-hybrid analysis using hTectonin as bait and the three isoforms of ficolins (L-, H- and M-ficolin) as prey. Results showed that the hTectonin (clone QZ7L1) interacts specifically with M-ficolin (GenBank Accession No. O00602) (Figure 4B). M-ficolin has in turn been shown to interact with the CRP [26]. Since both the CRP and M-ficolin are key proteins of the complement classical and lectin pathways, respectively, this is the first evidence for the potential function of a human Tectonin domain-containing protein in frontline immune defense. Further delineation of hTectonin to isolate its functional domains showed that only the sub-clones expressing the predicted Tectonin domains interacted with M-ficolin. Furthermore, only the fibrinogen-like (FBG) domain of M-ficolin was shown to interact with the hTectonin, concurring with recent findings that the FBG domain is responsible for ligand-binding [12]. These results suggest that the protein-protein interaction between the *hypothetical* hTectonin and M-ficolin is not random, but structurally and positionally specific, and that the hTectonin is potentially involved in immune regulation, acting through its Tectonin domains.

### Tectonin domains harbor high avidity LPS-binding motifs

**Figure 5:**
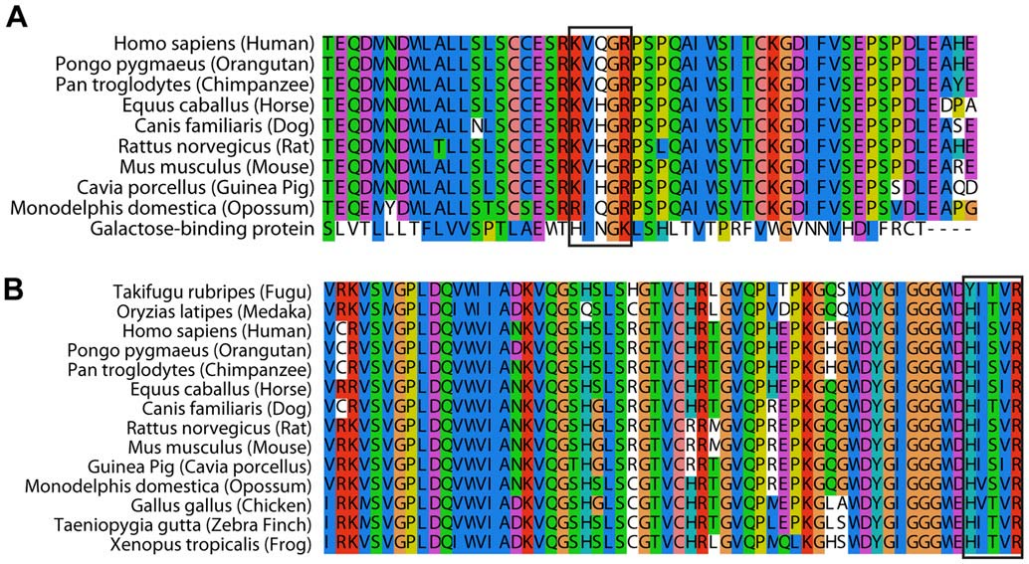
The LPS-binding motifs of hTectonin are conserved in other species. The LPS-binding motif of the pattern BHB(P)HB (blue box) in - (A) hTect6 and (B) hTect11 - are well-conserved in other species.

**Figure 6:**
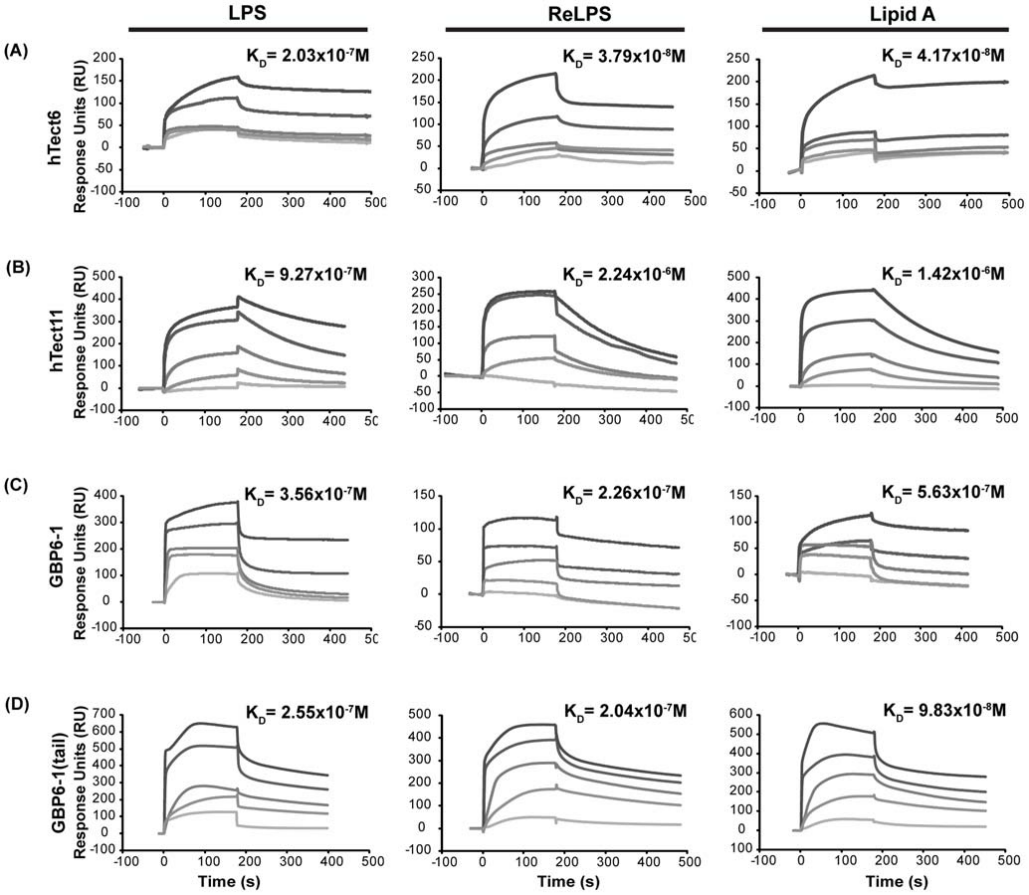
hTectonin peptides and GBP peptides bind LPS, ReLPS and lipid A with high afiinity. hTectonin peptides, namely hTec6 and hTec11, containing the predicted LPS-binding motif [27], [28] are able to bind the bacterial endotoxin. In GBP, the exclusion of the C-terminal tail loop which is not part of the β-propeller Tectonin structure (Supporting Figure S1B) - GBP6-1– gives similar binding affinity with the peptide designed to include this tail region, showing that it does not play an important role in the binding to lipid A, and that the function comes from within the Tectonin domains. Control peptides derived from non-Tectonin regions showed no binding to LPS (see Supporting Figure S1C), thus confirming the specificity of interaction with LPS via the Tectonin domains.

Gram negative bacterial endotoxin or lipopolysaccharide (LPS) is a prominent and well-studied representative pathogen-associated molecular pattern. Proteins harboring LPS-binding motifs, with alternating basic-hydrophobic/polar residues (BHB(P)HB), have been shown to bind LPS via the lipid A moiety [27], [28], which is the most conserved bioactive pathophysiological centre of the LPS molecule (Supporting Figure S1A). Based on the BHB(P)HB pattern, we identified two such motifs in the 6^th^ and 11^th^ Tectonin domains of the hTectonin and found that these motifs were well-conserved among the mammalian homologs of hTectonin in addition to being in a region of high sequence conservation (Figure 5). Representative Tectonin peptides were synthesized around the BHB(P)HB motifs in Tectonin domains 6 & 11, and their efficacy of binding of lipid A was compared with peptides derived from the GBP Tectonin domains 1 & 6 (Supporting Figure S1B), where similar BHB(P)HB motifs exist. Real-time biointeraction of these Tectonin peptides to lipid A immobilized on biacore HPA chip showed that indeed the hTectonin peptides bound the lipid A at affinities of K_D_ 10^−7 to −8^ M, which are similar to the GBP peptides (Figure 6 and Table 1). We also showed that both the hTectonin and the GBP peptides exhibited similar level of binding affinity to ReLPS and LPS (Figure 6 and Supporting Figure S1 A,C). Table 1 summarises and compares the binding affinities of various peptides derived from the hTectonin and GBP. This corroborates our hypothesis and demonstrates the pathogen-binding ability of Tectonin domains and its functional conservation across species, from horseshoe crab GBP to human hTectonin.

**Table 1:** Dissociation constants of Tectonin peptides when bound to LPS, ReLPS, lipid A.

Bacterial ligand	Peptide	Sequence (LPS-binding motif underlined)	K_D_ (mol^−1^)
LPS	GBP6-1(tail)	KSCWLNPFLAEWTHINGKLSH	2.55×10^−7^
	GBP6-1	FESVPASKAEWTHINGKLSH	3.56×10^−7^
	hTectonin6	LSLSCCESRKVQGRPSPQAI	2.03×10^−7^
	hTectonin11	IGGGWDHISVRANATRAPRS	9.27×10^−7^
ReLPS	GBP6-1(tail)		2.04×10^−7^
	GBP6-1		2.26×10^−7^
	hTectonin6		3.79×10^−8^
	hTectonin11		2.24×10^−6^
Lipid A	GBP6-1(tail)		9.83×10^−8^
	GBP6-1		5.63×10^−7^
	hTectonin6		4.17×10^−8^
	hTectonin11		1.42×10^−6^

## Discussion

In order to classify and complete the functional characterization of the human proteome, many of the unknown proteins are usually inferred from their counterparts in other species. This seems to be an easy option if the proteins share high sequence similarity, as they can be matched to each other by performing a simple sequence matching. However, the task is more complicated if the proteins do not show homology in their primary sequences. Nevertheless, many related proteins show conserved functionality more in terms of domain and structural conservation.

In this paper, we report our discovery of a human Tectonin protein, hitherto classified as being hypothetical. By structure-function analyses, we inferred its function as an immune-related protein. We showed that similar to its invertebrate counterparts, the hTectonin protein functions via its Tectonin domains. Furthermore, a distance PSI-BLAST sequence matching indicates that although the hTectonin shows low sequence homology, it is phylogenetically related to known proteins with Tectonin domains, functioning as immune proteins. By SMART domain comparison, we show that hTectonin contains multiple homologs widespread in the vertebrate kingdom, implying that it is not a one-off protein in the human proteome, but rather, an important one conserved throughout many species. We also discovered that the hTectonin gene is expressed in the human leukocytes. This is interesting, as a recent addition to the human database of proteins showed another human leukocyte Tectonin protein called the leukolectin (GenBank Accession No. ACM77812.1) [29], which also exhibits five Tectonin domain repeats (Figure 1B). This further implicates hTectonin to be immune-related. Like its limulus counterpart, GBP, which interacts with an important complement initiator (CRP), we find that the hTectonin also interacts with a cognate complement lectin, Ficolin. Furthermore, this interaction is specific, involving only the Tectonin domains within the hTectonin protein. We also identified LPS-binding motifs within two of the Tectonin domains which are located in the highly conserved sequence of the β-propeller fold. The affinity of these motifs for bacterial LPS and the truncated active forms of the endotoxin molecule (ReLPS and lipid A) was verified experimentally. Thus, we propose that hTectonin is a novel human protein that forms a β-propeller structure which is involved in protein-protein interaction with immune-related proteins such as ficolin, and it simultaneously interacts with pathogens via PAMPs like LPS. Thus, the hTectonin plays a vital role in immune defense, which is conserved over a vast number of organisms.

## Materials and Methods

### Identification of Tectonin proteins

Tectonin domain containing proteins were identified using domain search on the SMART database [22], [23]. A position-specific iterated search using the primary sequence on PSI-Search on the EMBL server was performed using GBP as the query sequence. Related sequences were chosen after 2 iterations of PSI-Search. Hits were put through the SMART prediction server to confirm their propensity to form Tectonin domains. Multiple sequence alignment was carried out on the curated list of proteins using Promals3D [30]. A phylogenetic tree was then constructed from sequences showing strong domain alignments using PHYLIP [31] with a bootstrap value of 1000.

### Human hTectonin cDNA clones

The hTectonin (Q7Z6L1) cDNA was obtained from iDNA OpenBiosystems (MHS1010-9205594). The cDNA was subcloned into pGBKT7 and pGADT7 vectors for the yeast 2-hybrid experiments.

### Yeast-2-hybrid assay for protein-protein interaction

Co-transformations of the different bait and prey plasmids into *S. cerevisiae* AH109 strain were performed in accordance to [16]. The full-length and subclones of hTectonin and the ficolin cDNAs (without their signal sequences) were each fused to the DNA-binding domain of Gal4 in the bait plasmid pGBKT7 (BD Biosciences), or to the activation domain of Gal4 in the prey plasmid pGADT7-Rec (BD Biosciences). For selection, synthetic complete (SC) media lacking Leu and Trp (SC-Trp-Leu) or lacking Leu, Trp, His and adenine (quadruple dropout, QDO medium) were used. Transformants containing bait and prey plasmids were selected on SC-Trp-Leu by incubation for 3.5 days at 30°C. Resulting colonies were suspended in water and replated on SC-Trp-Leu and QDO agar at 30°C. The negative control was co-transformed with a recombinant plasmid and an empty prey or bait plasmid. The positive control was co-transformed with a plasmid expressing the full-length Gal4 transcriptional activator together with the empty pGADT7-Rec vector. Positive transformants were selected in SC media lacking Trp. hTectonin plasmid in pGBKT7 was then transformed into the library-positive yeast. DNA from resulting colonies from the co-transformation on QDO agar were extracted and identified through sequencing.

### Peptide design and synthesis

The hTectonin protein sequence was scanned for LPS-binding motif. Two potential sites with the BHPHB pattern were found in hTectonin domains 6 (KVQGR) and 11 (HISVR). Henceforth, these peptides are referred to as hTec peptides (hTec6 and hTec11). The hTec peptide length and region surrounding the LPS-binding motif was chosen and optimized based on hydrophilicity and solubility values. The h-Tec6 was: LSLSCCESRKVQGRPSPQAI and hTec11 was IGGGWDHISVRANATRAPRS. For comparison, one BHPHB site was found in the limulus GBP, Tectonin domain 1 (HINGK). The GBP peptides are: GBP6-1(tail) KSCWLNPFLAEWTHINGKLSH and GBP6-1(no tail) FESVPASKAEWTHINGKLSH, which are annotated based on the amino acid residues which encompass the domains 6-to-1. Peptides were also designed from the combination of GBP Tectonin domains 1 and 6. The peptides were synthesized by Genemed Synthesis, Inc., USA, and purified to >95% under pyrogen-free conditions.

### Surface plasmon resonance analysis of the peptides

Surface plasmon resonance analysis for real-time biointeraction between the Tectonin peptides and bacterial LPS was performed using a Biacore 2000 instrument (Biacore AB). LPS, ReLPS and lipid A from *Salmonella minnesota* (List Biologicals, UK) were diluted to 0.25 mg/ml in 20 mM sodium phosphate, 150 mM NaCl, pH 7.4 and immobilized on the surface of an HPA sensor chip (Biacore AB) according to the manufacturer's specifications. Binding of the Tectonin peptides to the immobilized ligands was measured at a flow rate of 20 µl/min in 10 mM Tris, 150 mM NaCl, pH 7.4. Regeneration of the chip surface was achieved by injection of 20 µl 0.1 M NaOH until steady baseline was achieved. The dissociation constant, KD was calculated using BiaEvaluation software, version 3.2.

## Supporting Information

Figure S1 (A) Structure of the bacterial LPS. LPS structure and the truncated forms, ReLPS and lipid A. (B) The structure of GBP, with the tail (circled) at the C-terminal end, which does not form the β-propeller structure of GBP. (C) Control Tectonin peptides which do not harbor the LPS-binding motif of BHPHB do not bind lipid A.
(0.24 MB TIF)

## References

[1] Human Genome Project Information. Oak Ridge, Tennessee: U.S. Department of Energy Office of Science, Office of Biological and Environmental Research

[2] Huh CG, Aldrich J, Mottahedeh J, Kwon H, Johnson C (1998). Cloning and characterization of Physarum polycephalum tectonins. Homologues of Limulus lectin L-6.. J Biol Chem.

[3] Schroder HC, Ushijima H, Krasko A, Gamulin V, Thakur NL (2003). Emergence and disappearance of an immune molecule, an antimicrobial lectin, in basal metazoa. A tachylectin-related protein in the sponge Suberites domuncula.. J Biol Chem.

[4] Kawabata S, Tsuda R (2002). Molecular basis of non-self recognition by the horseshoe crab tachylectins.. Biochim Biophys Acta.

[5] Galliano M, Minchiotti L, Campagnoli M, Sala A, Visai L (2003). Structural and biochemical characterization of a new type of lectin isolated from carp eggs.. Biochem J.

[6] Kawabata S, Iwanaga S (1999). Role of lectins in the innate immunity of horseshoe crab.. Dev Comp Immunol.

[7] Basak S, Banerjee R, Mukherjee I, Das S (2009). Influence of domain architecture and codon usage pattern on the evolution of virulence factors of Vibrio cholerae.. Biochem Biophys Res Commun.

[8] Bogdanov YF, Grishaeva TM, Dadashev SY (2007). Similarity of the domain structure of proteins as a basis for the conservation of meiosis.. Int Rev Cytol.

[9] Fulop V, Jones DT (1999). Beta propellers: structural rigidity and functional diversity.. Curr Opin Struct Biol.

[10] Jawad Z, Paoli M (2002). Novel sequences propel familiar folds.. Structure.

[11] Yadid I, Tawfik DS (2007). Reconstruction of functional beta-propeller lectins via homo-oligomeric assembly of shorter fragments.. J Mol Biol.

[12] Zhang J, Koh J, Lu J, Thiel S, Leong BS (2009). Local inflammation induces complement crosstalk which amplifies the antimicrobial response.. PLoS Pathog.

[13] Chen SC, Yen CH, Yeh MS, Huang CJ, Liu TY (2001). Biochemical properties and cDNa cloning of two new lectins from the plasma of Tachypleus tridentatus: Tachypleus plasma lectin 1 and 2+.. J Biol Chem.

[14] Chiou ST, Chen YW, Chen SC, Chao CF, Liu TY (2000). Isolation and characterization of proteins that bind to galactose, lipopolysaccharide of Escherichia coli, and protein A of Staphylococcus aureus from the hemolymph of Tachypleus tridentatus.. J Biol Chem.

[15] Kuo TH, Chuang SC, Chang SY, Liang PH (2006). Ligand specificities and structural requirements of two Tachypleus plasma lectins for bacterial trapping.. Biochem J.

[16] Le Saux A, Ng PM, Koh JJ, Low DH, Leong GE (2008). The macromolecular assembly of pathogen-recognition receptors is impelled by serine proteases, via their complement control protein modules.. J Mol Biol.

[17] Iwanaga S (2002). The molecular basis of innate immunity in the horseshoe crab.. Curr Opin Immunol.

[18] Ponting CP, Mott R, Bork P, Copley RR (2001). Novel protein domains and repeats in Drosophila melanogaster: insights into structure, function, and evolution.. Genome Res.

[19] Saito T, Kawabata S, Hirata M, Iwanaga S (1995). A novel type of limulus lectin-L6. Purification, primary structure, and antibacterial activity.. J Biol Chem.

[20] Iwanaga S, Lee BL (2005). Recent advances in the innate immunity of invertebrate animals.. J Biochem Mol Biol.

[21] Mali B, Soza-Ried J, Frohme M, Frank U (2006). Structural but not functional conservation of an immune molecule: a tachylectin-like gene in Hydractinia.. Dev Comp Immunol.

[22] Letunic I, Copley RR, Pils B, Pinkert S, Schultz J (2006). SMART 5: domains in the context of genomes and networks.. Nucleic Acids Res.

[23] Schultz J, Milpetz F, Bork P, Ponting CP (1998). SMART, a simple modular architecture research tool: identification of signaling domains.. Proc Natl Acad Sci U S A.

[24] Jones DT (1999). Protein secondary structure prediction based on position-specific scoring matrices.. J Mol Biol.

[25] Bryson K, McGuffin LJ, Marsden RL, Ward JJ, Sodhi JS (2005). Protein structure prediction servers at University College London.. Nucleic Acids Res.

[26] Ng PM, Le Saux A, Lee CM, Tan NS, Lu J (2007). C-reactive protein collaborates with plasma lectins to boost immune response against bacteria.. EMBO J.

[27] Frecer V, Ho B, Ding JL (2000). Interpretation of biological activity data of bacterial endotoxins by simple molecular models of mechanism of action.. Eur J Biochem.

[28] Frecer V, Ho B, Ding JL (2004). De novo design of potent antimicrobial peptides.. Antimicrob Agents Chemother.

[29] Miftari MH, Walther BT (2009). Molecular cloning and characterization of the leukolectin gene isolated from the human leukocytes.

[30] Pei J, Kim BH, Grishin NV (2008). PROMALS3D: a tool for multiple protein sequence and structure alignments.. Nucleic Acids Res.

[31] Retief JD (2000). Phylogenetic analysis using PHYLIP.. Methods Mol Biol.

[32] Thompson JD, Gibson TJ, Higgins DG (2002). Multiple sequence alignment using ClustalW and ClustalX.. Curr Protoc Bioinformatics.

[33] Waterhouse AM, Procter JB, Martin DM, Clamp M, Barton GJ (2009). Jalview Version 2–a multiple sequence alignment editor and analysis workbench.. Bioinformatics.

