# Supplementary figures and images for: A Novel Human Tectonin Protein with Multivalent β-Propeller Folds Interacts with Ficolin and Binds Bacterial LPS
Source: PLoS One. 2009 Jul 16;4(7):e6260. doi: 10.1371/journal.pone.0006260 (PMC2707011; doi:10.1371/journal.pone.0006260)

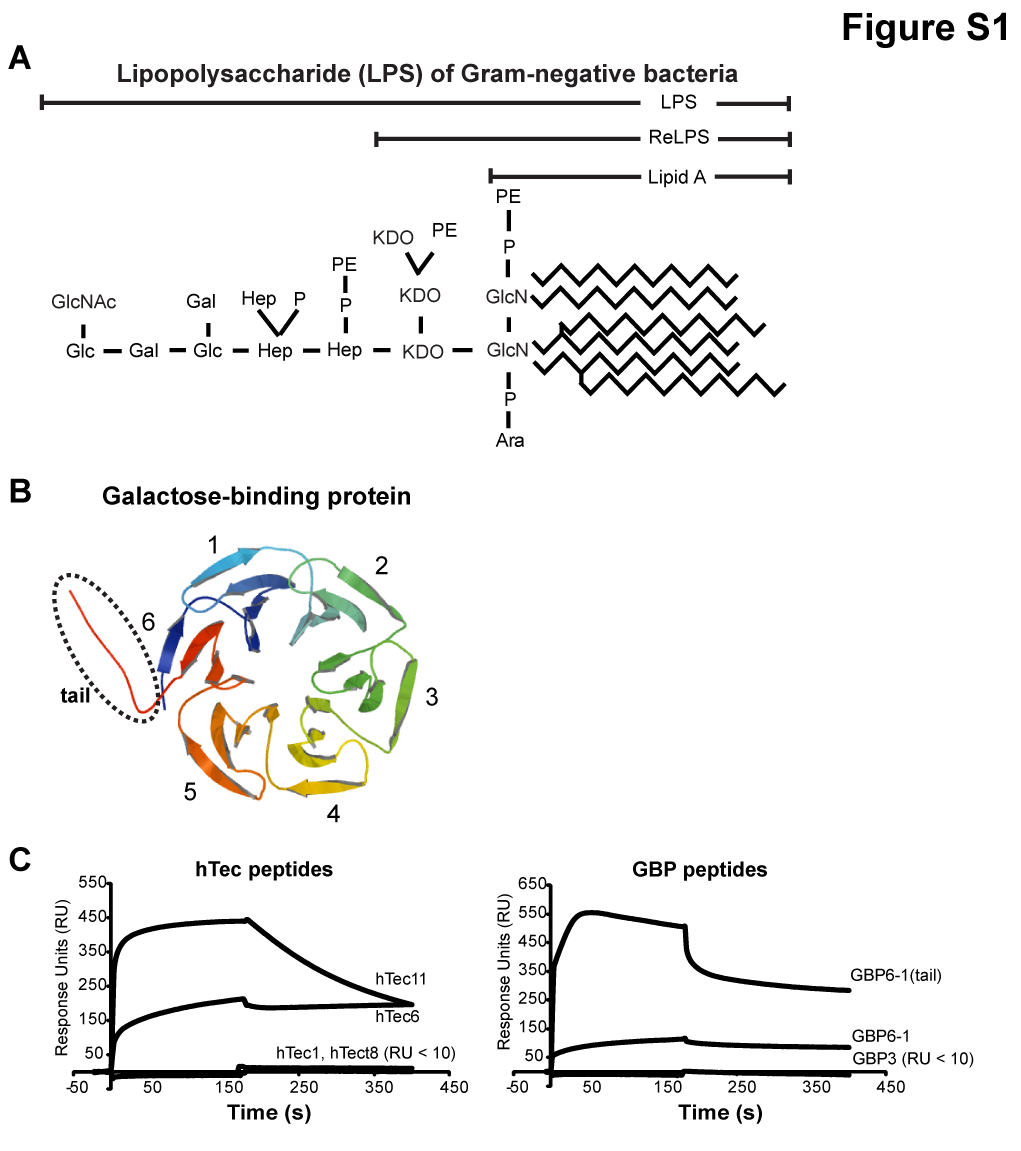

Supplement: Figure S1 — (A) Structure of the bacterial LPS. LPS structure and the truncated forms, ReLPS and lipid A. (B) The structure of GBP, with the tail (circled) at the C-terminal end, which does not form the β-propeller structure of GBP. (C) Control Tectonin peptides which do not harbor the LPS-binding motif of BHPHB do not bind lipid A. (0.24 MB TIF) [file pone.0006260.s001.tif]
